# Unfolded protein response inhibits KAT2B/MLKL-mediated necroptosis of hepatocytes by promoting BMI1 level to ubiquitinate KAT2B

**DOI:** 10.1515/med-2023-0718

**Published:** 2023-06-16

**Authors:** Xiaogang Huang, Xiongzhi He, Rongxian Qiu, Xuemei Xie, Fengfeng Zheng, Feihua Chen, Zhenting Hu

**Affiliations:** Department of Infectious Diseases, The Affiliated Hospital of Putian University, Putian City, Fujian Province, 351100, China; Department of Infectious Diseases, The Affiliated Hospital of Putian University, No. 999 Dongzhen East Road, Licheng District, Putian City, Fujian Province, 351100, China

**Keywords:** hepatocytes, necroptosis, unfolded protein response, B cell-specific Moloney MLV insertion site-1, KAT2B

## Abstract

Unfolded protein response (UPR) plays an important role in the pathogenesis of many liver diseases. BMI1 has a liver protection effect, but whether it participates in the regulation of hepatocyte death through UPR is not well defined. Herein, the endoplasmic reticulum stress model was established by inducing hepatocyte line (MIHA) with tunicamycin (TM, 5 µg/ml). Cell counting kit-8 assay and flow cytometry were used to evaluate the viability and apoptosis of hepatocytes. The expression levels of BMI1, KAT2B, and proteins related to UPR (p-eIF2α, eIF2α, ATF4, and ATF6), NF-κB (p65 and p-p65), apoptosis (cleaved caspase-3, bcl-2, and bax) and necroptosis (p-MLKL and MLKL) were determined by Western blot. The relationship between KAT2B and BMI1 was determined by co-immunoprecipitation and ubiquitination assay. The results showed that TM not only promoted UPR, apoptosis, and necroptosis in hepatocytes but also upregulated the expression levels of BMI1 and KAT2B and activated NF-κB pathway. BAY-117082 reversed the effects of TM on viability, apoptosis, NF-κB pathway, and BMI1 but strengthened the effects of TM on KAT2B/MLKL-mediated necroptosis. BMI1 promoted the ubiquitination of KAT2B, and BMI1 overexpression reversed the effects of TM on viability, apoptosis, and KAT2B/MLKL-mediated necroptosis. In summary, overexpression of BMI1 promotes the ubiquitination of KAT2B to block the MLKL-mediated necroptosis of hepatocytes.

## 1 Introduction

The liver is the largest biological metabolic organ in the body, and hepatocytes are the main cells to maintain the function and shape of the liver. Due to the particularity of the anatomical location and function, the liver is vulnerable to viruses, exogenous substances, and endogenous metabolites, resulting in damaged hepatocytes, which is the common pathophysiological basis of assorted liver lesions, and the main cause of liver dysfunction [1,2]. A previous study has found that irreversible damage of hepatocytes will lead to hepatocyte death, including apoptosis and necroptosis, which is a key event in the progression of liver disease [3]. Therefore, improving the anti-injury ability of hepatocytes, reducing hepatocyte death, and promoting injury repair are important strategies for alleviating and preventing liver failure.

The accumulated evidence indicates that endoplasmic reticulum (ER) stress plays an important role in the pathogenesis of many liver diseases [4]. ER is a key organelle for the synthesis of protein and lipids. When the homeostasis of ER is disturbed, misfolded protein will accumulate, leading to ER stress [5]. ER stress will trigger the unfolded protein response (UPR) to restore ER homeostasis [6]. UPR can prevent the synthesis of protein in ER and promote the degradation of protein through activating transcription factor 6 (ATF6) and phosphorylated eukaryotic translational initiation factor 2 alpha (eIF2α), thus promoting cell survival [7]. Tian et al. reported that the phosphorylation of eIF2α mitigates ER stress and hepatocyte necroptosis in acute liver injury [8]. However, more mechanisms by which UPR reduces hepatocyte death remain to be elucidated.

In our preliminary study, we found that UPR upregulated the protein expression of B cell-specific Moloney MLV insertion site-1 (BMI1) in the hepatocytes, which might be related to the activation of NF-κB pathway induced by UPR [9], as this pathway has been proved to upregulate BMI1 level [10]. BMI1 is a key transcription inhibitor that regulates gene expression during hematopoietic development [11]. Also, a prior research has reported that the loss of BMI1 can cause liver damage, so BMI1 may play a role in liver protection [12]. In addition, through bioinformatics analysis, we predicted that BMI1 is the ubiquitination-modified protein of lysine acetyltransferase 2B (KAT2B). KAT2B has been shown to mediate mixed lineage kinase domain-like pseudokinase (MLKL)-dependent necroptosis [13]. However, it is unclear whether the mechanism of UPR protecting hepatocytes is implicated in the regulation of KAT2B/MLKL-mediated necroptosis. To this end, this study probed into the relationship between UPR-mediated BMI1 upregulation and KAT2B/MLKL-mediated necroptosis during ER stress in hepatocytes.

## 2 Materials and methods

### 2.1 Cell culture

Human normal hepatocytes MIHA (CL0469; Fenghuishengwu, China) were maintained in RPMI-1640 medium (IMC-202; Immocell, China) supplemented with 10% fetal bovine serum (FBS, IMC-101; Immocell) and Penicillin-Streptomycin Solution (IMC-601; Immocell) at 37°C under the humidified air with 5% CO_2_.

### 2.2 Bioinformatics analysis

The interaction between KAT2B and BMI1 was predicted by ubibrowser (http://ubibrowser.ncpsb.org.cn).

### 2.3 Transfection

BMI1 overexpression plasmid (oe-BMI1, F105834) and its negative control (NC, pcDNA3.1-3xFlag) were purchased from YouBio (China). The BMI1-specific short hairpin RNA (shBMI1, target sequences: ATTGATGCCACAACCATAATA) and the empty vector (shNC) were ordered from VectorBuilder (China). To achieve transfection, the transfection reagent (L3000150; ThermoFisher, USA) and plasmid or shRNA were separately diluted with Opti-MEM (11058021; ThermoFisher), mixed, and then added to the cells for incubation. Following 24 h, the cells were subjected to quantitative reverse transcription polymerase chain reaction (qRT-PCR) to verify the transfection efficiency.

### 2.4 Grouping

This study was divided into three parts. In the first part, the cells were treated with Tunicamycin (TM, 5 µg/ml, HY-A0098; MedChemExpress, China) for 0, 12, 24, and 48 h, and then, the cell viability was assayed by cell counting kit-8 (CCK-8) [14]. TM treatment for 48 h was selected for the subsequent experiment to evaluate the effect of TM on MIHA cells. In the second part, cells were pretreated with NF-κB inhibitor BAY-117082 (10 µM, HY-13453; MedChemExpress) for 1 h and then received TM (5 µg/ml) treatment for 0, 12, 24, and 48 h, subsequent to which the cell viability was measured by CCK-8 [15]. In the subsequent experiments, cells were pretreated with BAY-117082 for 1 h and then treated with TM for 48 h. In the third part, the cells transfected with oe-BMI1, NC, shNC, or shBMI1 were further treated with TM (5 µg/ml) for 0, 12, 24, and 48 h, whose viability was then tested by CCK-8. In subsequent experiments, the transfected cells underwent 48 h of TM treatment.

### 2.5 Cell viability

CCK-8 kit (K1018, Apexbio, China) was applied to evaluate the viability of treated MIHA cells. Briefly, cells were inoculated in a 96-well plate (3,000 per well). After 24 h of incubation, the cells were treated in different ways for different times and then reacted with CCK-8 reagent for 2 h. Lastly, the absorbance at 450 nm was detected by a microplate reader (CMaxPlus, MD, China).

### 2.6 Western blot

Whole-cell lysates were prepared by RIPA lysis buffer (YT612; Bjbalb, China). Total proteins were quantified by BCA kit (BTN80815; Bjbalb), loaded onto SDS-PAGE electrophoresis, and later transferred to nitrocellulose membranes (P2110; Applygen, China). Afterwards, nonspecific binding was blocked with 5% skim milk, and the membranes were incubated with primary antibodies at 4°C overnight and probed with secondary antibodies (Table 1) at room temperature. Glyceraldehyde-3-phosphate dehydrogenase (GAPDH) was used as an internal reference. Eventually, immunoreactions were detected by ECL reagent (CW0048; CWBIO, China) and visualized in the ChemiDoc™ XRS plus imaging System (Bio-Rad, USA).

**Table 1:** Antibodies used in this study

Name	Catalog	Molecular weight (kDa)	Dilution	Manufacturer
p-eIF2α	#3398	38	1/1,000	Cell Signaling Technology, USA
eIF2α	#5324	38	1/1,000	Cell Signaling Technology, USA
ATF4	ab184909	50	1/1,000	Abcam, UK
ATF6	ab122897	75	1/1,000	Abcam, UK
P65	ab32536	65	1/1,000	Abcam, UK
p-p65	ab76302	65	1/1,000	Abcam, UK
BMI1	ab126783	40	1/10,000	Abcam, UK
KAT2B	ab12188	93	1/1,000	Abcam, UK
p-MLKL	ab187091	54	1/1,000	Abcam, UK
MLKL	ab184718	54	1/1,000	Abcam, UK
Bcl-2	ab32124	26	1/1,000	Abcam, UK
Bax	ab32503	21	1/1,000	Abcam, UK
Cleaved caspase-3	ab32042	17	1/500	Abcam, UK
GAPDH	ab8245	36	1/10,000	Abcam, UK
Goat anti-rabbit	ab205718	—	1/2,000	Abcam, UK
Goat anti-mouse	ab205719	—	1/2,000	Abcam, UK

### 2.7 Cell apoptosis

Annexin V-FITC/PI kit (abs5001; absin, China) was employed to analyze the apoptotic cells. In a nutshell, the treated MIHA cells were gently suspended in the binding buffer to prepare cell suspension with the concentration of 1 × 10^6^ cells/ml. Thereafter, Annexin V-FITC and propidium iodide were sequentially added to the cell suspension for 20 min of reaction. Ultimately, the apoptotic cells were detected by a flow cytometer (NL-3000; CYTEK, USA).

### 2.8 qRT-PCR

Total RNA was isolated from MIHA cells using RNA Extraction Reagent (19201ES60; YEASEN, China). Then, the isolated RNA was reversely transcribed into cDNA using a cDNA Synthesis Kit (11119ES60; YEASEN). Subsequently, qPCR was performed by SYBR Green qPCR Mix (11198ES03; YEASEN) in a real-time PCR system (ABI 7300; ABI, USA). The primer sequences are listed below: BMI1-forward: 5′-AGATCGGGGCGAGACAATG-3′, reverse: 5′-TTTTATTCTGCGGGGCTGGG-3′; GAPDH-forward: 5′-GTCTCCTCTGACTTCAACAGCG-3′, reverse: 5′-ACCACCCTGTTGCTGTAGCCAA-3′. The expression values were normalized to GAPDH using the 2^−ΔΔCT^ method [16].

### 2.9 Co-Immunoprecipitation (co-IP)

The interaction between BMI1 and KAT2B was verified by co-IP kit (Bes3011; BersinBio, China). Briefly, cell lysates were prepared in RIPA buffer and centrifuged at 13,000 × *g* for 10 min to obtain the supernatant. Part of the supernatant was utilized as input and the remaining was used for IP. The protein was then incubated with anti-KAT2B antibody (Table 1) or anti-IgG antibody (in the co-IP kit) overnight. Thereafter, the proteins were immunoprecipitated by Protein A/G beads and the precipitated proteins were subjected to the detection of Western blot.

### 2.10 Ubiquitination assay

The ubiquitination level of KAT2B was determined by Ubiquitination detection Kit (BK161; Cytoskeleton, USA) as previously described [17]. In short, cells transfected with shNC or shBMI1 were treated with 20 μM MG132 (HY-13259; MedChemExpress) for 4 h and then lysed with lysis buffer. Subsequently, the lysate was incubated with ubiquitination affinity bead suspension. The beads were washed with washing buffer and samples were eluted. Subsequent standard Western blot procedures were performed using the indicated antibodies.

### 2.11 Statistical analysis

The measurement data were presented as mean ± standard deviation. One-way analysis of variance was adopted for comparison among multiple groups, followed by post hoc Bonferroni. Two groups were compared through independent-samples *t*-test. All statistical analyses were implemented with Graphpad 8.0 software (California, USA), and *P*-values less than 0.05 were considered to be statistically significant.

## 3 Results

### 3.1 TM induced apoptosis and necroptosis of MIHA cells

**Figure 1:**
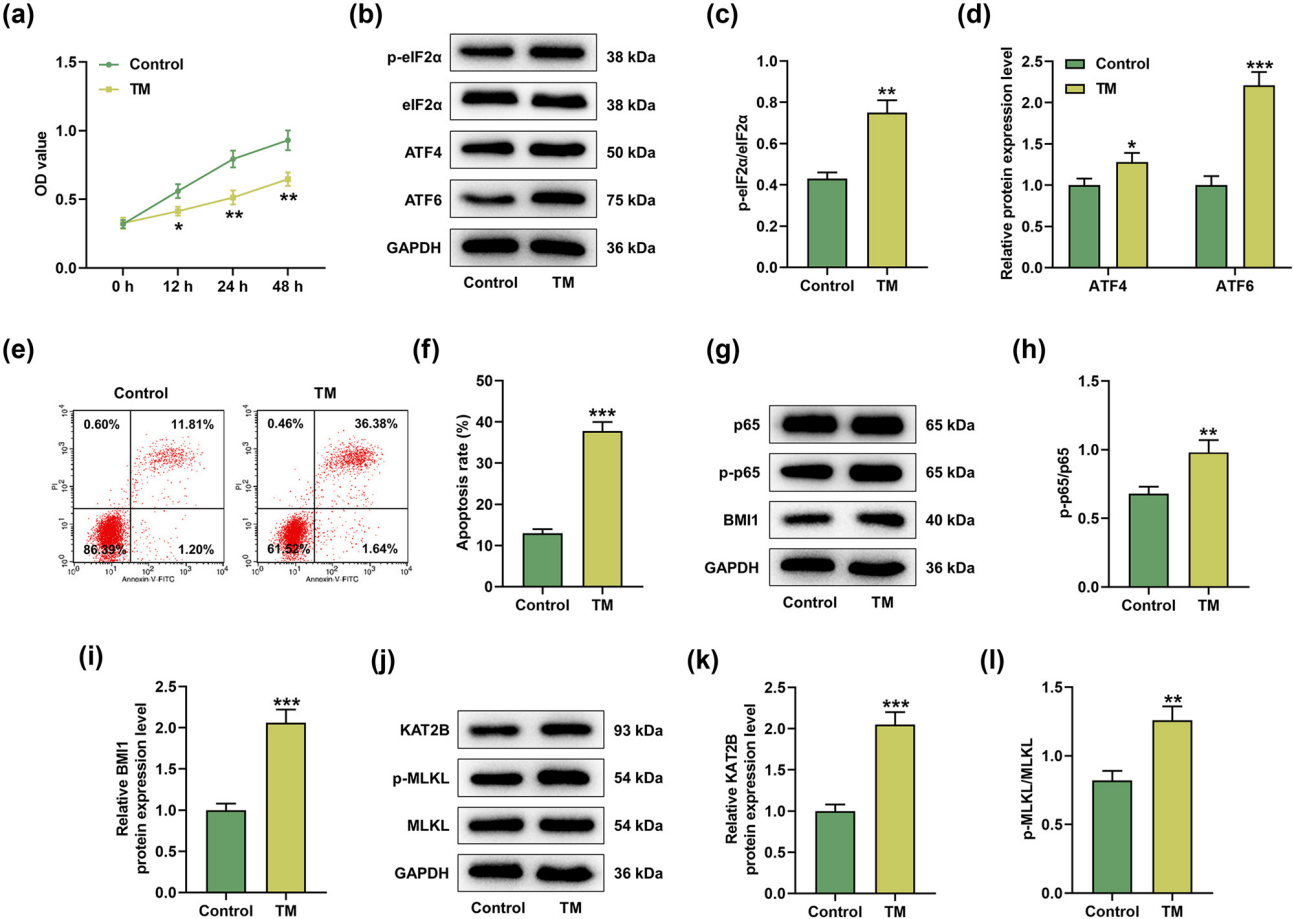
TM induced apoptosis and necroptosis of MIHA cells. (a) The viability of MIHA cells treated with or without TM (5 µg/ml) was analyzed at 0, 12, 24, and 48 h by CCK-8 assay. (b–d) The protein levels of p-eIF2α, eIF2α, ATF4, and ATF6 in MIHA cells treated with or without TM (5 µg/ml) were determined by Western blot. (e and f) The apoptosis of MIHA cells treated with or without TM (5 µg/ml) was determined by flow cytometry. (g–i) The protein levels of p65, p-p65, and BMI1 in MIHA cells treated with or without TM (5 µg/ml) were assayed by Western blot. (j–l) The protein levels of KAT2B, p-MLKL, and MLKL in MIHA cells treated with or without TM (5 µg/ml) were tested by Western blot. GAPDH was used as the internal control. ^*^ *P* < 0.05, ^**^ *P* < 0.01, ^***^ *P* < 0.001 vs control. Quantified values were presented as mean ± standard deviation of at least three independent experiments. TM: Tunicamycin. CCK-8: cell counting kit-8. GAPDH: glyceraldehyde-3-phosphate dehydrogenase.

After 12, 24, and 48 h of TM treatment, the cell viability was significantly reduced (Figure 1a, *P* < 0.05). To further evaluate the effect of TM on MIHA cells, MIHA cells underwent TM treatment for 48 h. We detected the effect of TM treatment on the expression levels of UPR-related proteins in MIHA cells by Western blot, and the results unveiled that TM increased the ratio of p-eIF2α/eIF2α as well as the protein levels of ATF4 and ATF6 (Figure 1b–d, *P* < 0.05). Furthermore, TM enhanced the apoptosis rate of MIHA cells (Figure 1e and f, *P* < 0.001). Next, we found that TM also promoted p-p65/p65 and p-MLKL/MLKL ratios and upregulated the protein expression levels of BMI1 and KAT2B in MIHA cells (Figure 1g–l, *P* < 0.01). These evidences indicated that TM could promote the activation of NF-κB pathway and KAT2B/MLKL pathway and upregulate the expression of BMI1 in MIHA cells.

### 3.2 NF-κB inhibitor blocked TM-induced apoptosis but enhanced TM-induced necroptosis

**Figure 2:**
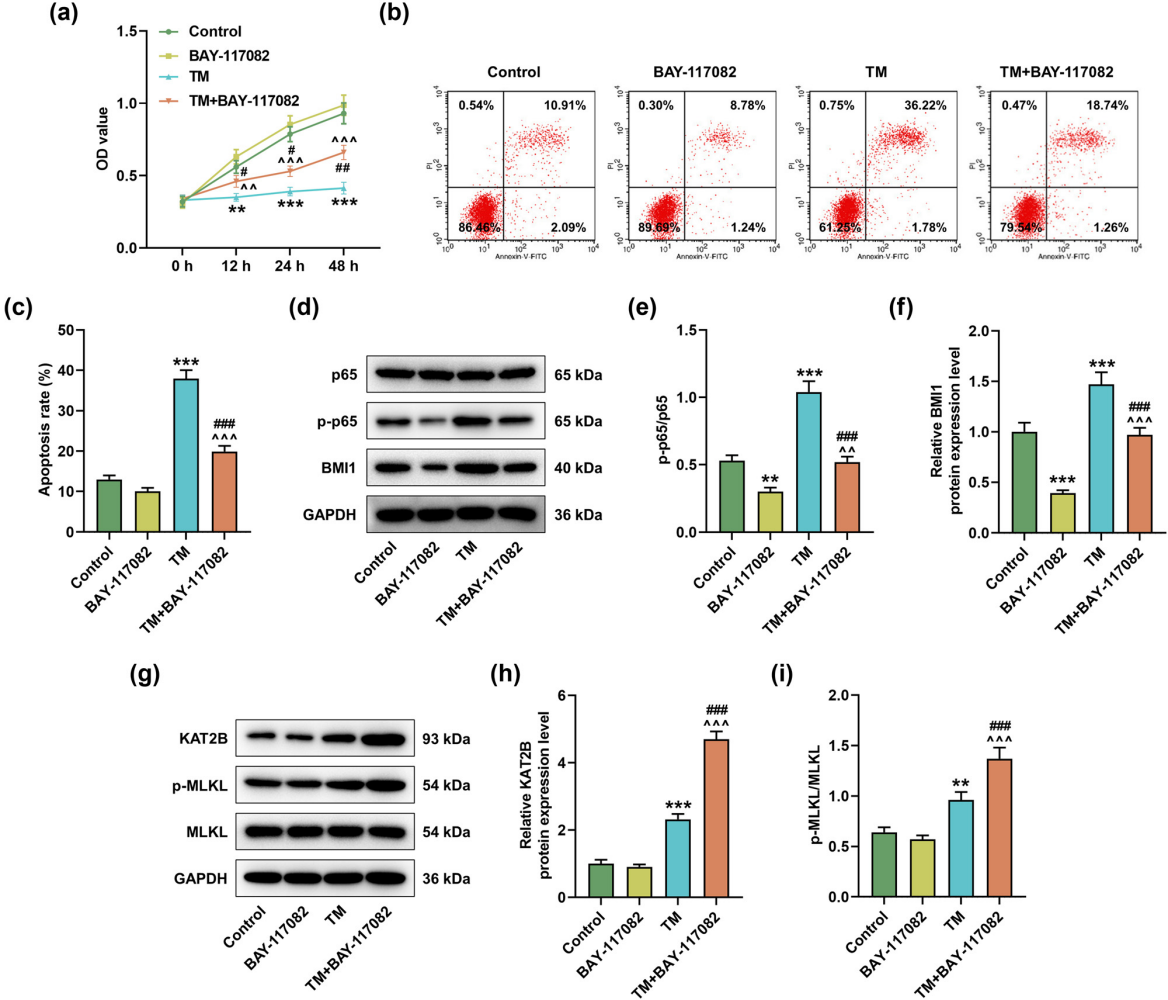
TM induced apoptosis and necroptosis of MIHA cells through NF-κB pathway. The experiment was divided into four groups: cells in the control group were cultured normally; cells in the BAY-117082 group were treated with BAY-117082 (10 µM) for 1 h; cells in the TM group were treated with TM (5 µg/ml) for different times; and cells in the TM + BAY-117082 group were pretreated with BAY-117082 (10 µM) for 1 h and then treated with TM (5 µg/ml) for different times. (a) The viability of treated MIHA cells was analyzed at 0, 12, 24, and 48 h by CCK-8 assay. (b and c) The apoptosis of treated MIHA cells was analyzed by flow cytometry. (d–f) The protein levels of p65, p-p65, and BMI1 in treated MIHA cells were determined by Western blot. (g–i) The protein levels of KAT2B, p-MLKL, and MLKL in treated MIHA cells were examined by Western blot. GAPDH was applied as the internal control. ^**^ *P* < 0.01, ^***^ *P* < 0.001 vs control. ^^^^ *P* < 0.01, ^^^^^ *P* < 0.001 vs BAY-117082. ^#^ *P* < 0.05, ^###^ *P* < 0.001 vs TM. Quantified values were presented as mean ± standard deviation of at least three independent experiments. TM: Tunicamycin. CCK-8: cell counting kit-8. GAPDH: glyceraldehyde-3-phosphate dehydrogenase.

To verify whether the regulation of MIHA cells by TM was related to the NF-κB pathway, MIHA cells were pretreated with the NF-κB inhibitor BAY-117082 and then treated with TM. The results showed that BAY-117082 alone had no significant effect on cell viability and apoptosis, but it partially reversed the regulation of TM on cell viability and apoptosis (Figure 2a–c, *P* < 0.05). Furthermore, BAY-117082 decreased the p-p65/p65 ratio and BMI1 protein level and also reversed the regulation of TM on p-p65/p65 ratio and BMI1 protein expression (Figure 2d–f, *P* < 0.01). Interestingly, BAY-117082 did not significantly affect KAT2B expression level and p-MLKL/MLKL ratio, but it enhanced the promoting effect of TM on KAT2B expression level and p-MLKL/MLKL ratio (Figure 2g–i, *P* < 0.001).

### 3.3 BMI1 overexpression reversed the effects of TM on apoptosis and necroptosis

**Figure 3:**
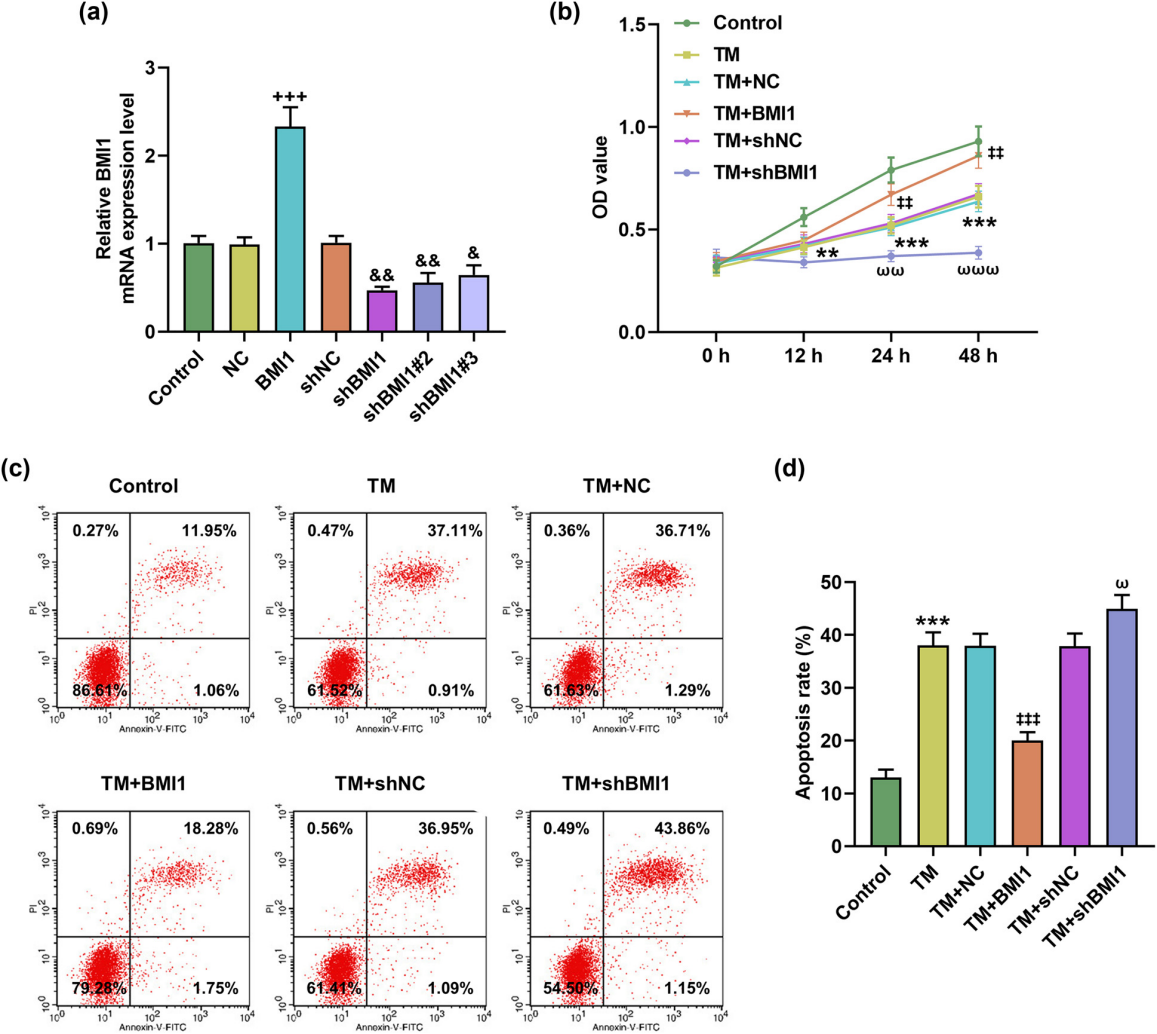
BMI1 overexpression reversed the effects of TM on the viability and apoptosis of MIHA cells. (a) The transfection efficiency of BMI1 overexpression plasmid, shBMI1, shBMI1#2, and shBMI1#3 was determined by qRT-PCR. GAPDH was employed as the internal control. (b–d) MIHA cells were transfected with NC, BMI1 overexpression plasmid, shBMI1, or shNC and then were treated with TM (5 µg/ml) for different times. (b) The viability of treated MIHA cells was analyzed at 0, 12, 24, and 48 h by CCK-8 assay. (c and d) The apoptosis of treated MIHA cells was analyzed by flow cytometry. ^+++^ *P* < 0.001 vs NC. ^**^ *P* < 0.01, ^***^ *P* < 0.001 vs control. ^&^ *P* < 0.05, ^&&^ *P* < 0.01 vs shNC. ^‡‡^ *P* < 0.01, ^‡‡‡^ *P* < 0.001 vs TM + NC. ^ω^ *P* < 0.05, ^ωω^ *P* < 0.01, ^ωωω^ *P* < 0.001 vs TM + shNC. Quantified values were presented as mean ± standard deviation of at least three independent experiments. ShBMI1: BMI1-specific short hairpin RNA. NC: negative control. GAPDH: glyceraldehyde-3-phosphate dehydrogenase. TM: Tunicamycin. CCK-8: cell counting kit-8. qRT-PCR: quantitative reverse transcription polymerase chain reaction.

**Figure 4:**
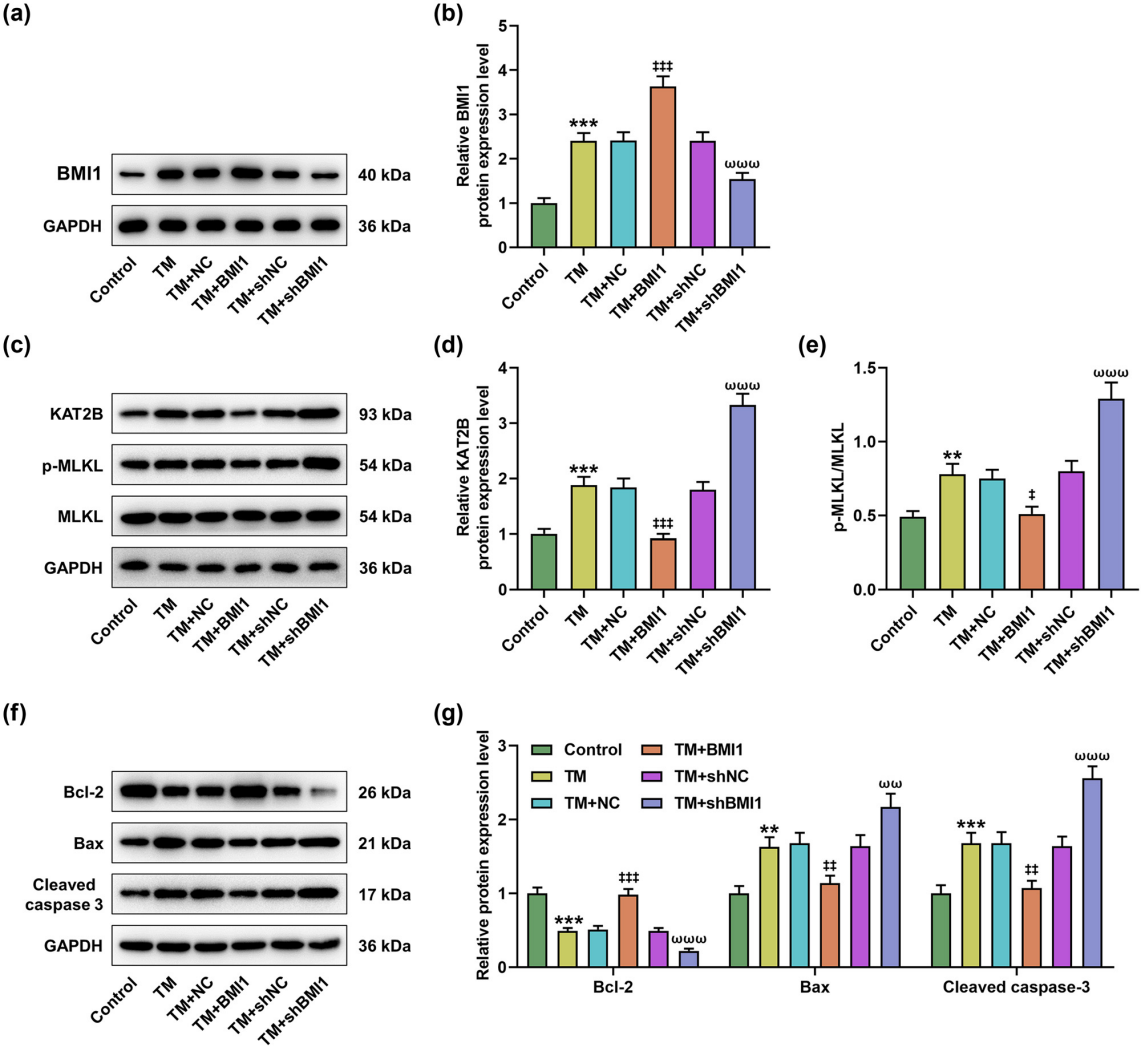
BMI1 overexpression reversed the effects of TM on KAT2B/MLKL pathway-related proteins and apoptosis-related proteins in MIHA cells. MIHA cells were transfected with NC, BMI1 overexpression plasmid, shBMI1, or shNC and then were treated with TM (5 µg/ml) for 48 h. (a–e) The protein levels of BMI1, KAT2B, p-MLKL, and MLKL in treated MIHA cells were determined by Western blot. (f and g) The expression levels of apoptosis-related proteins (Bcl-2, Bax, and cleaved caspase-3) were determined by Western blot. GAPDH was exploited as the internal control. ^**^ *P* < 0.01, ^***^ *P* < 0.001 vs control. ^‡^ *P* < 0.05, ^‡‡^ *P* < 0.01, ^‡‡‡^ *P* < 0.001 vs TM + NC. ^ωωω^ *P* < 0.001 vs TM + shNC. Quantified values were presented as mean ± standard deviation of at least three independent experiments. ShBMI1: BMI1-specific short hairpin RNA. NC: negative control. GAPDH: glyceraldehyde-3-phosphate dehydrogenase. TM: Tunicamycin.

To determine whether the effect of TM on MIHA cells is related to BMI1, we overexpressed and silenced BMI1 in MIHA cells. As per the detection results of qRT-PCR, shBMI1 presented the most obvious transfection efficiency among shBMI1, shBMI1#2, and shBMI1#3 (Figure 3a, *P* < 0.01). Also, it was found that oe-BMI1 reversed the effects of TM on the viability and apoptosis of MIHA cells, while shBMI1 further potentiated the inhibiting or promoting the impact of TM upon cell viability or apoptosis (Figure 3b–d, *P* < 0.05). In addition, the protein level of BMI1 increased by TM was further strengthened by oe-BMI1 but was reversed by shBMI1 (Figure 4a and b, *P* < 0.001). Furthermore, KAT2B protein level and p-MLKL/MLKL ratio regulated by TM were also reversed by oe-BMI1 but were strengthened by shBMI1 (Figure 4c–e, *P* < 0.05). Through the detection of apoptosis-related proteins, we found that TM inhibited the expression of Bcl-2, but increased the levels of Bax and cleaved caspase-3 (Figure 4f and g, *P* < 0.01). Similarly, the effects of TM on apoptosis-related proteins were also negated by oe-BMI1 and strengthened by shBMI1 (Figure 4f and g, *P* < 0.01).

### 3.4 MLKL knockdown reversed the effects of TM on apoptosis and necroptosis

**Figure 5:**
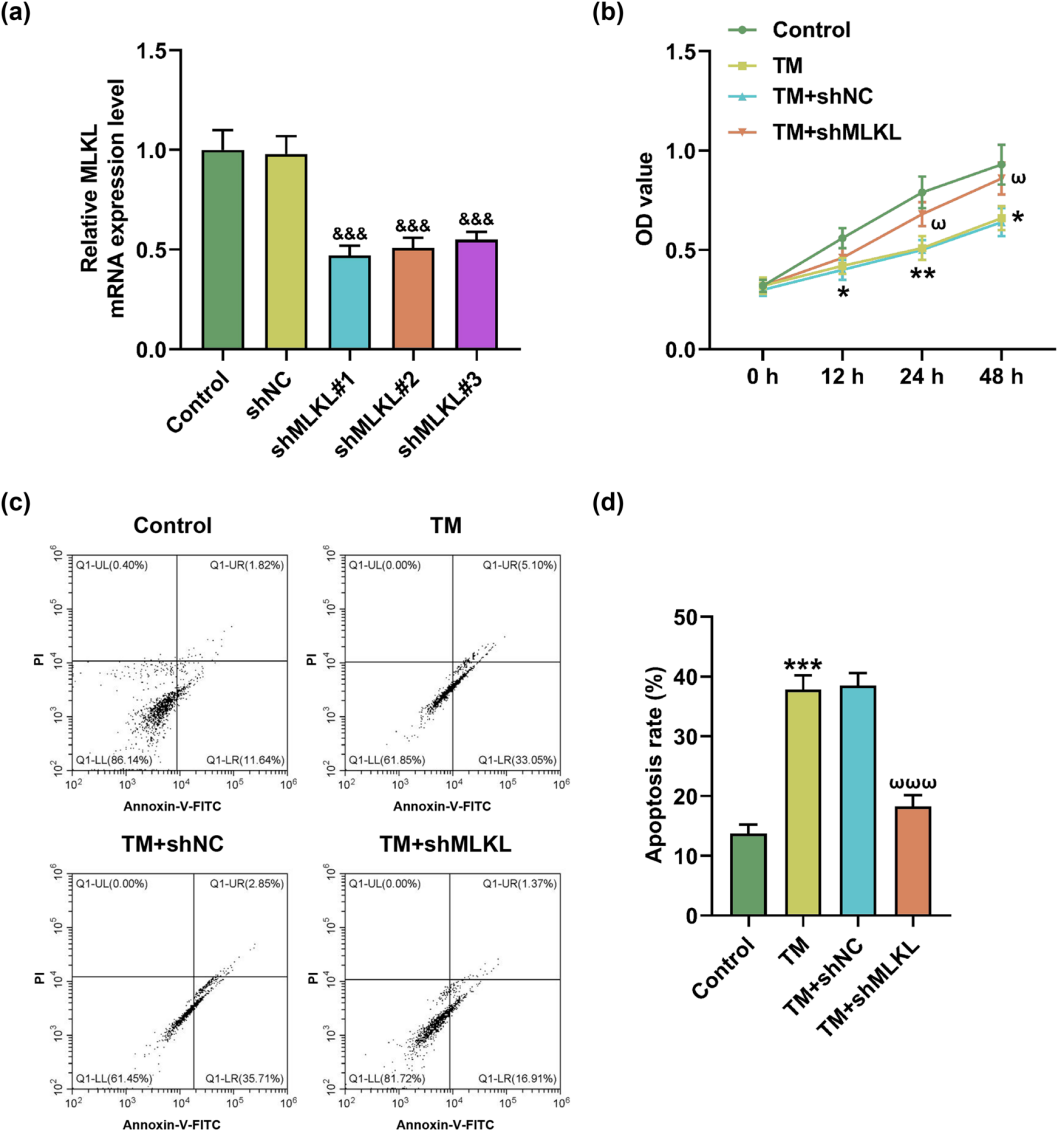
MLKL knockdown reversed the effects of TM on viability and apoptosis of MIHA cells. (a) The transfection efficiency of shMLKL, shMLKL#2, and shMLKL#3 was determined by qRT-PCR. GAPDH was employed as the internal control. (b) The viability of treated MIHA cells was analyzed at 0, 12, 24, and 48 h by CCK-8 assay. (c and d) The apoptosis of treated MIHA cells was analyzed by flow cytometry. ^&&&^ *P* < 0.001 vs shNC. ^*^ *P* < 0.05, ^**^ *P* < 0.01, ^***^ *P* < 0.001 vs control. ^ω^ *P* < 0.05, ^ωωω^ *P* < 0.001 vs TM + shNC. Quantified values were presented as mean ± standard deviation of at least three independent experiments. MLKL: mixed lineage kinase domain-like pseudokinase. ShMLKL: MLKL-specific short hairpin RNA. shNC: shRNA negative control. GAPDH: glyceraldehyde-3-phosphate dehydrogenase. TM: Tunicamycin. CCK-8: cell counting kit-8. qRT-PCR: quantitative reverse transcription polymerase chain reaction.

**Figure 6:**
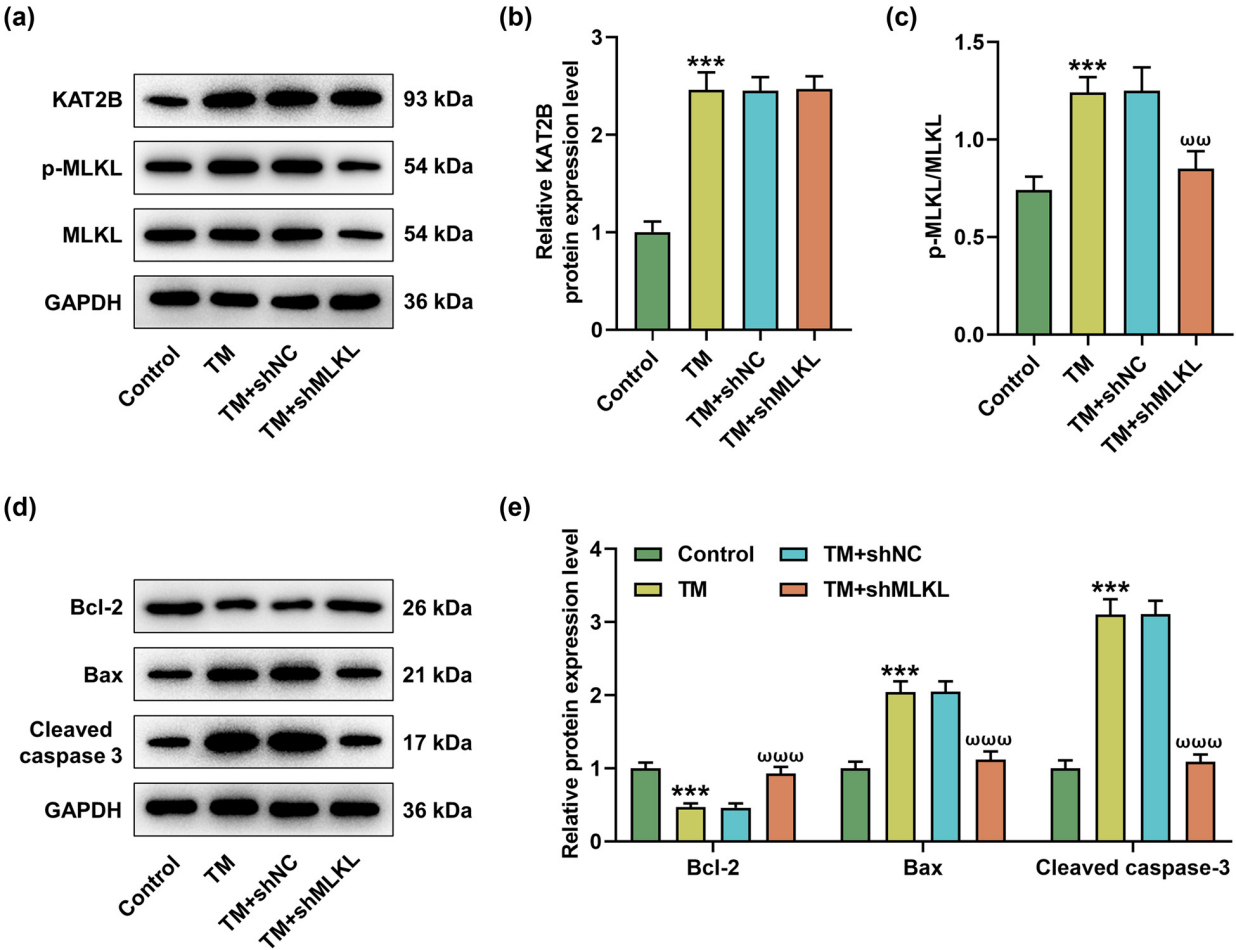
MLKL knockdown reversed the effects of TM on apoptosis-related proteins in MIHA cells. (a–c) The protein levels of KAT2B, p-MLKL, and MLKL in treated MIHA cells were determined by Western blot. (d–e) The expression levels of apoptosis-related proteins (Bcl-2, Bax, and cleaved caspase-3) were determined by Western blot. GAPDH was exploited as the internal control. ^***^ *P* < 0.001 vs control. ^ωω^ *P* < 0.01, ^ωωω^ *P* < 0.001 vs TM + shNC. Quantified values were presented as mean ± standard deviation of at least three independent experiments. ShMLKL: MLKL-specific short hairpin RNA. NC: negative control. GAPDH: glyceraldehyde-3-phosphate dehydrogenase. TM: Tunicamycin.

To investigate whether MLKL plays a leading role in BMI1/KAT2B-involved necroptosis, shMLKL transfection, and TM treatment were performed in MIHA cells. Besides, the most prominent transfection efficiency of shMLKL was verified by qRT-PCR (Figure 5a, *P* < 0.001). Notably, MLKL knockdown reversed the effects of TM on the viability (Figure 5b, *P* < 0.05) and apoptosis (Figure 5c and d, *P* < 0.001) of MIHA cells. However, MLKL knockdown did not affect the effects of TM on the protein level of KAT2B but reduced p-MLKL/MLKL ratio (Figure 6a–c, *P* < 0.001). Furthermore, we found that TM inhibited the expression of Bcl-2 but promoted the expression levels of Bax and cleaved caspase-3 (Figure 6d and e, *P* < 0.001). Similarly, the effects of TM on apoptosis-related proteins (Bcl-2, Bax, and cleaved caspase-3) were also neutralized by MLKL knockdown (Figure 6d and e, *P* < 0.01).

### 3.5 BMI1 affected the ubiquitination of KAT2B in MIHA cells

**Figure 7:**
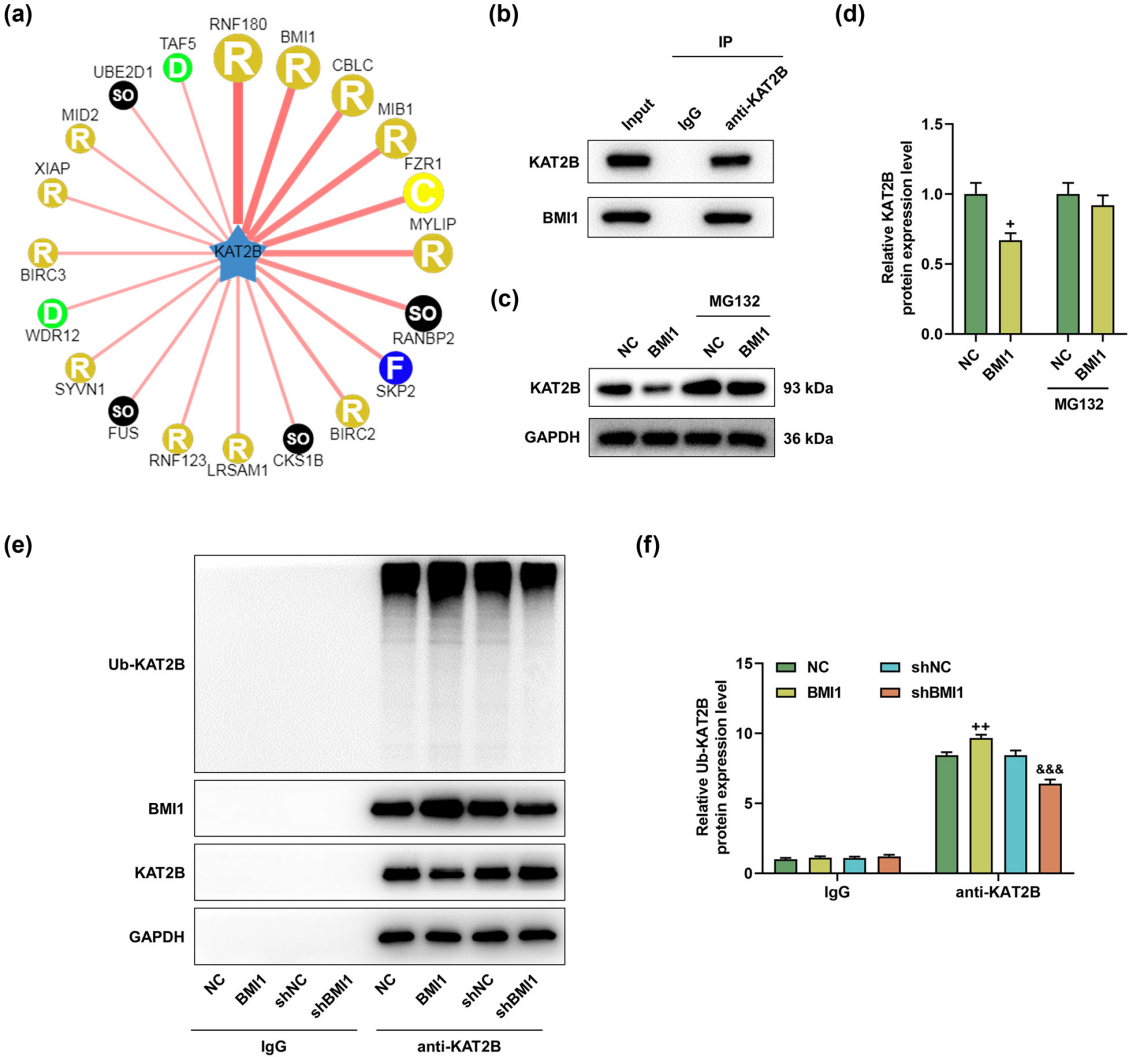
BMI1 mediated the ubiquitination of KAT2B. (a) The interaction between KAT2B and BMI1 was predicted by ubibrowser. R, RING (Really Interesting New Gene); C, C-terminal SOCS box; SO, single other; F, F-box; D, DWD box. The width of the red edge reflects the confidence of the interaction. (b) The interaction between KAT2B and BMI1 was determined by co-IP assay. (c and d) MIHA cells were transfected with NC or BMI1 overexpression plasmid and then were treated with 20 μM MG132 for 4 h. The protein level of KAT2B was determined by Western blot. (e and f) MIHA cells were transfected with shNC or shBMI1. After 24 h, the ubiquitination level of KAT2B was detected by ubiquitination assay. ^+^ *P* < 0.05, ^++^ *P* < 0.01 vs NC; ^&&&^ *P* < 0.001 vs shNC. NC: negative control. GAPDH: glyceraldehyde-3-phosphate dehydrogenase. shBMI1, BMI1-specific short hairpin RNA. shNC, negative control of shBMI1.

The interaction between KAT2B and BMI1 was predicted by ubibrowser (Figure 7a) and subsequently confirmed by co-IP experiments (Figure 7b). In addition, Western blot results showed that oe-BMI1 inhibited the protein expression of KAT2B (Figure 7c and d, *P* < 0.05). After treatment with protease inhibitor MG132, the inhibitory effect of oe-BMI1 on KAT2B was found to be blocked (Figure 7c and d). Moreover, BMI1 silencing increased the expression level of KAT2B but decreased the ubiquitination level of KAT2B, whereas BMI1 overexpression generated opposite results (Figure 7e and f, *P* < 0.01). Therefore, BMI1 affected the ubiquitination of KAT2B in MIHA cells.

## 4 Discussion

In this study, we found that UPR in hepatocytes protect hepatocytes by upregulating BMI1 level to ubiquitinate KAT2B, thereby blocking MLKL-mediated necroptosis.

TM is a natural nucleoside antibiotic, which can hinder the glycosylation modification of new proteins in ER and mediate cell apoptosis. Multiple studies induce ER stress in hepatocytes by TM [18,19]. As a previous study illustrated, TM-induced ER stress results in decreased hepatocyte viability and increased apoptosis [20]. In addition, TM can upregulate the expression levels of proteins related to UPR and necroptosis in hepatocytes [21]. Consistent with previous studies, we found that TM not only promoted UPR, apoptosis, and necroptosis in hepatocytes but also upregulated the expression levels of BMI1 and KAT2B and activated NF-κB pathway.

The activation of the NF-κB pathway induced by TM was due to crosstalk between the UPR and NF-κB pathway. UPR is synergistically mediated by IRE1, ATF6, and PERK. In addition, activated IRE1, activated PERK, and eIF2α phosphorylation have been revealed to activate NF-κB [22]. NF-κB can promote ER stress by upregulating the transcription and activity of protein in JNK pathway or by promoting the expression levels of inflammatory mediators [23,24]. Fu et al. proposed that ER stress in diabetic cardiomyopathy cells can be effectively reduced by inhibiting NF-κB signal [25]. Here, we reported that inhibition of NF-κB signal effectively reversed not only the regulation of TM on the vitality and apoptosis of MIHA cells but also the regulation of TM on BMI1, indicating that the increase in BMI1 expression was related to the activation of NF-κB induced by TM, which was also analogous to a previous report [10]. Interestingly, we found that inhibition of NF-κB signal promoted the regulation of KAT2B and p-MLKL by TM, signifying that blocking NF-κB could promote necroptosis of hepatocytes. This is actually not novel, as a previously published study has elucidated that inhibition of NF-κB can lead to RIPK1-mediated necroptosis in keratinocytes [26]. Since the inhibition of BMI1 promotes the occurrence of necroptosis, blocking NF-κB-induced necroptosis in hepatocytes may be related to the blocking of NF-κB-induced inhibition of BMI1 [27].

Existing research indicates that BMI1 helps maintain the self-renewal characteristics of normal stem cells and cancer cells [27,28]. In addition, BMI1 is an important gene for the expansion of hepatic progenitor cells [29], which indicates that BMI1 is also of great significance for the self-renewal of liver. Through loss- and gain-of-function assays, we found that oe-BMI1 inhibited TM-induced apoptosis and necroptosis of MIHA cells, while shBMI1 was the opposite, suggesting that BMI1 was the key gene to regulate MIHA cell apoptosis and necroptosis. This conclusion is also supported by the existing research. For instance, BMI1 can inhibit apoptosis and promote the survival of auditory hair cells by regulating oxidative stress and mitochondrial function [30]. Yuan et al. pinpointed that upregulation of BMI1 can inhibit the expression of cleaved caspase-3 yet promote the expression of Bcl-2, thus inhibiting hepatocyte apoptosis and reducing liver lipotoxicity [31]. Barabino et al. unraveled that the absence of BMI1 could lead to necroptosis in the retina of mice, thereby affecting retinal development [32]. These evidences collectively mirror that BMI1 protects hepatocytes from ER stress by inhibiting apoptosis and necroptosis.

This study also clarified the interaction between BMI1 and KAT2B. Specifically, BMI1 promoted the ubiquitination of KAT2B. Notably, BMI1 binds to the catalyzed RING2/RING1b subunit to form a functional E3 ubiquitin ligase that promotes protein degradation through the proteasome degradation pathway [33,34]. Although there is no research on ubiquitination of KAT2B by BMI1, KAT2B has been proved to be ubiquitinated by UBE2D2 [35]. Since KAT2B was able to mediate MLKL-dependent necroptosis, BMI1 might inhibit necrptosis in MIHA cells by promoting the ubiquitination of KAT2B, which was consistent with the result that oe-BMI1 reversed the promoting impacts of TM upon KAT2B and p-MLKL. Of note, TM-induced increases in BMI1 and KAT2B in MIHA cells were not inconsistent with the effect of BMI1 on the ubiquitination of KAT2B. Previous studies reported that KAT2B is helpful to maintain the effective UPR level in β cells [36]. Therefore, in TM-induced hepatocytes, the ubiquitination regulation of KAT2B by BMI1 is not enough to offset the promotion of KAT2B expression by other pathways. Hence, promoting the expression of BMI1 may be contributive to improving TM-induced ER stress.

Although this study clarified the potential mechanism of UPR on hepatocyte death, there is still a limitation, namely the exclusive use of the human normal hepatocyte line. Furthermore, the present findings *in vitro* need to be further validated in appropriate animal experiments in the future.

## 5 Conclusions

This study demonstrates that UPR activates the NF-κB pathway to promote BMI1 expression, which leads to the ubiquitination of KAT2B and thus blocks MLKL-mediated necroptosis. The present findings shed new insights into the underlying molecular mechanisms that control liver diseases.
